# Nerve conduction studies through landmark based recording and effects of hand length

**DOI:** 10.1038/s41598-023-36967-8

**Published:** 2023-06-20

**Authors:** Sai Deepak Yaranagula, Venkata Krishna Chaitanya Koduri, P. Suresh Babu, Thriveni Veligonda

**Affiliations:** Department of Neurology, AIG Hospitals, 136, Plot No 2/3/4/5 Survey, 1, Mindspace Road, Gachibowli, Hyderabad, Telangana 500032 India

**Keywords:** Neuroscience, Physiology, Neurology

## Abstract

As per existing guidelines, the distance between stimulator and recording electrodes in nerve conduction studies (NCS) should be the same (fixed) in all the subjects, i.e., it should not be based on anatomical landmarks. However, there are no studies comparing fixed distance recordings with landmark based NCS. We postulated that hand length can influence the NCS parameters in fixed distance recordings and this can be nullified using landmark based recordings. To test this theory, we performed NCS in 48 normal subjects as per standard guidelines (standard protocol) and then compared it to NCS with ulnar styloid as the landmark (modified protocol). NCS were performed on median and ulnar nerves of the right upper limb. Three motor NCS parameters including distal latency, compound muscle action potential (CMAP) amplitudes and nerve conduction velocities were measured. Sensory nerve action potential (SNAP) amplitudes and conduction velocities were the two sensory parameters measured. On analysis, ulnar motor conduction velocity was the only parameter affected by hand length in both standard and modified protocols. Modified protocol did not have any additional advantage to the standard protocol advised by NDTF. We conclude that the NDTF guidelines are therefore reasonable when considering the effects of hand length. Possible reasons for this result including anatomical and anthropometric explanations are discussed.

## Introduction

The normative data task force (NDTF) has stipulated that recording of nerve conduction studies (NCS) are best done by maintaining a fixed distance between the stimulator and recording electrodes (fixed distance recordings) rather than landmark based recordings^1^. We hypothesised that, in fixed distance recordings, hand length can significantly alter normative data, as patients with longer hands will have a relatively more distal stimulation/recording and vice versa. We also hypothesised that this variation can be nullified using a landmark based NCS. To test these hypotheses, we performed NCS in normal healthy volunteers. NCS in each subject were first performed with a standard protocol of fixed distance between stimulator and recording electrode as per NDTF guidelines. We then used a modified protocol in which stimulation (in motor NCS) and recording (in sensory NCS) was done 1 cm proximal to the ulnar styloid along the course of the median and ulnar nerves. Median and Ulnar Compound muscle action potential (CMAP) parameters (distal latency, distal amplitude, conduction velocity) and Sensory nerve action potentials (SNAP) parameters (amplitude, conduction velocity) with respect to variation in hand length were compared in both the standard and modified protocol.

## Materials and methods

### Inclusion and exclusion criteria

NCS were done on 48 normal healthy volunteers after written informed consent. Study protocol was approved by the hospital ethics committee AIG/IEC.I]H&R 16 (h)/07.2021-09. All methods were performed in accordance with the relevant guidelines and regulations. Consenting adults above age of 18 years were included in the study. Exclusion criteria were history of diabetes/hypertension, known thyroid illness, known vitamin B12 deficiency or history of malignancy, presence of weakness, numbness or paresthesias in any part of body, previous nerve surgery, excessive alcohol intake operationally defined as more than once a week, and previously diagnosed carpal tunnel syndrome.

### Anthropometric measurements

Anthropometric measurements were done using standard equipment. Weight was recorded with digital weighing machine (EQUINOX Model EQ-EB-9300). Height was recorded with Stadiometer (Indosurgicals Model 20015). Tips of radial and ulnar styloids were marked. The line connecting the tips of radial and ulnar styloids was designated inter-styloid line. Midpoint of inter-styloid line was marked as mid styloid point. Hand length was measured with segmometer (CESCORF 211537) between the tip of middle finger and mid styloid point. Digital thermometer (Omron ModelMC-720) was used to measure temperature in mid palm and dorsum of foot and maintained at or above 32C in all the subjects.

### Nerve conduction studies

All the NCS were done by a single trained Neurophysiology technologist. NCS were done on Natus Elite-Synergy, Version: 22.4.0,122, with the following baseline recording settings: sensitivity: 5 mV/div (motor), 20 μV/div (sensory); sweep: 5 ms/div (motor), 2 ms/div (sensory); Low filter: 3 Hz; high filter: 10 kHz. Motor and sensory NCS were performed on the right median and right ulnar nerves. In the standard protocol, fixed distance of 80 mm between the stimulator and recording electrode for CMAP analysis and 140 mm for SNAP analysis was used. After the completion of the standard protocol, CMAP and SNAP recordings were done in the same subject using a modified protocol. In the modified protocol, stimulation (in CMAP) and recording (in SNAP) was done 1 cm proximal to the ulnar styloid along the course of the median and ulnar nerves. This was achieved by drawing a line parallel to wrist crease 1 cm proximal to ulnar styloid, with the palm/hand in neutral position. The point of intersection of this line with the median and ulnar nerves was used as point of stimulation (in CMAP) and recording (in SNAP). All SNAPs were recorded using orthodromic technique with 2 cm fixed distance between recording and reference electrodes in all the subjects, in both standard and modified protocols. Rest of the electrode and stimulator positions were as per the guidelines given by Chen et al.^2^. Proximal Ulnar motor stimulation was done below elbow.

### Statistical analysis

The data for the study after editing for completeness and consistency was entered into MS EXCEL for further analysis. The continuous variables were expressed as mean and standard deviation (SD). The categorical variables were expressed as percentage frequency distribution. The effect of Hand length on NCS parameters were assessed by Analysis of covariance (ANCOVA) with adjustment made for effects of age, gender, height and BMI, all of which can individually affect the NCS parameters^3^. The statistical package for social sciences (SPSS 21st version) was used for the analysis. P value < 0.05 with two sided was considered as significant.

## Results

### Demographic characteristics

Mean age of the cohort was 36 ± 11 years (range 19–65 years). There were 34 males and 14 females. Mean height was 1.652 ± 0.11 m (range 1.342–1.816 m), mean weight 71.97 ± 12.3 kg (range 51–112.4 kg) and mean BMI was 26.49 ± 4.7 kg/m^2^ (range 18.83–40.38 kg/m^2^).

### Standard vs modified protocol

Table 1 compares the NCS parameters in standard and modified protocols. Stimulator to recording electrode distance was significantly different in all the four NCS (motor median {p value-0.01}, motor ulnar {p value-0.0001}, sensory median {p value-0.0001} and sensory ulnar {p value-0.0001}). Motor amplitudes were similar in both the protocols. Stimulator to recording electrode distance in sensory NCS was significantly lower in modified protocol leading to significantly higher sensory amplitudes.

**Table 1:** Comparison of NCS parameters in standard and modified protocols using students t test.

	Standard protocol	Modified protocol	P value
	Mean	Mean	
Motor			
Median			
Latency (ms)	3.12 ± 0.3	3.23 ± 0.3	0.075
Amplitude (mV)	11.89 ± 2.0	12.07 ± 2.2	0.675
Conduction velocity (m/s)	57.9 ± 3.5	58.44 ± 3.8	0.504
Stimulator to recording electrode distance (mm)	80 + 0.0	82.92 ± 8.4	**0.010**
Ulnar			
Latency (ms)	2.69 ± 0.3	2.41 ± 0.3	**0.0001**
Amplitude (mV)	10.83 ± 2.0	10.93 ± 2.0	0.807
Conduction velocity (m/s)	63.42 ± 5.8	60.88 ± 5.5	**0.030**
Stimulator to recording electrode distance (mm)	80 + 0.0	65.63 ± 5.3	**0.0001**
Sensory			
Median			
Amplitude (mcV)	16.44 ± 5.6	27.75 ± 8.5	**0.0001**
Conduction velocity (m/s)	54.41 ± 5.7	53.91 ± 4.6	0.637
Stimulator to recording electrode distance (mm)	140 + 0.0	129.75 ± 6.8	**0.0001**
Ulnar			
Amplitude (mcV)	14.54 ± 3.5	22.09 ± 6.7	**0.0001**
Conduction velocity (m/s)	62.76 ± 5.9	59.76 ± 5.9	**0.0001**
Stimulator to recording electrode distance (mm)	140 + 0.0	108.75 ± 7.9	**0.0001**

*ms* milliseconds, *mV* millivolts, *m/s* metre per second, *mcV* microvolts, *cm* centimetre.

Significant values are in bold.

### Shorter vs longer hands

In modified protocol, longer hands had significantly higher stimulator to recording electrode distance in all the four NCS (motor median {p value-0.039}, motor ulnar {p value-0.002}, sensory median {p value-0.001} and sensory ulnar {p value-0.0001}). Correspondingly, CMAP and SNAP amplitudes were lower. However, this difference in amplitudes reached statistical significance in only ulnar sensory NCS {p value-0.004} (Table 2).

**Table 2:** Comparison of NCS parameters in standard and modified protocols based on hand length using Mann Whitney U test.

	Standard Protocol		P value	Modified Protocol		P value
	Hand length184 and less	Hand length185 and more		Hand length184 and less	Hand length185 and more	
	n = 22	n = 26		n = 22	n = 26	
Motor						
Median						
Latency (ms)	3.09 ± 0.4	3.13 ± 0.2	0.218	3.16 ± 0.4	3.3 ± 0.3	**0.040**
Amplitude (mV)	12.39 ± 2.4	11.47 ± 1.5	0.082	12.65 ± 2.4	11.58 ± 1.9	0.068
Conduction velocity (m/s)	57.95 ± 3.9	57.85 ± 3.1	0.983	58.5 ± 4.7	58.38 ± 2.9	0.909
Stimulator to recording electrode distance (mm)	80 ± 0	80 ± 0	NA	80 ± 9.9	85.38 ± 6.2	**0.039**
Ulnar						
Latency (ms)	2.66 ± 0.3	2.72 ± 0.3	0.277	2.36 ± 0.3	2.45 ± 0.2	0.259
Amplitude (mV)	11.14 ± 2	10.56 ± 2	0.264	11.25 ± 2.1	10.66 ± 1.9	0.362
Conduction velocity (m/s)	63.41 ± 4.8	63.42 ± 6.7	0.844	63.05 ± 5.2	59.04 ± 5.2	**0.010**
Stimulator to recording electrode distance (mm)	80 ± 0	80 ± 0	NA	62.95 ± 5.5	67.88 ± 4	**0.002**
Sensory						
Median						
Amplitude (mcV)	16.73 ± 7.1	16.2 ± 4	0.717	30.71 ± 10.5	25.24 ± 5.3	0.717
Conduction velocity (m/s)	55.71 ± 6.6	53.32 ± 4.6	0.295	53.98 ± 4.9	53.85 ± 4.3	0.918
Stimulator to recording electrode distance (mm)	140 ± 0	140 ± 0	NA	126.59 ± 7.9	132.42 ± 4.3	**0.001**
Ulnar						
Amplitude (mcV)	14.78 ± 4	14.35 ± 3.1	0.508	25.04 ± 7.2	19.59 ± 5.1	**0.004**
Conduction velocity (m/s)	63.15 ± 5.7	62.43 ± 6.2	0.641	58.77 ± 5.4	60.6 ± 6.3	0.291
Stimulator to recording electrode distance (mm)	140 ± 0	140 ± 0	NA	103.41 ± 7	113.27 ± 5.5	**0.0001**

*ms* milliseconds, *mV* millivolts, *m/s* metre per second, *mcV* microvolts, *cm* centimetre, *NA* not available.

Significant values are in bold.

### Effects of hand length on NCS

The final effects of hand length on NCS in standard and modified protocols are tabulated in Table 3. A total of ten parameters were assessed. These included six motor (median and Ulnar distal latencies, CMAP amplitudes and conduction velocities), and four sensory (median and ulnar SNAP amplitudes and conduction velocities) parameters. Ulnar motor conduction velocities were the only parameter affected by hand length in both standard {p value-0.003} and modified protocol {p value-0.028}. None of the other nine parameters were influenced by hand length.

**Table 3:** Effects of Hand length on NCS in standard and modified protocols by ANCOVA (with adjustment made for effects of age, gender, height and BMI).

	Standard Protocol		Modified Protocol	
	F statistic	P value	F statistic	P value
Motor				
Median				
Latency	1.598	0.144	1.219	0.314
Amplitude	1.19	0.333	1.484	0.183
Conduction velocity	1.926	0.070	1.797	0.093
Ulnar				
Latency	0.712	0.728	0.663	0.772
Amplitude	0.686	0.751	0.752	0.692
Conduction velocity	3.352	**0.003**	5.345	**0.028**
Sensory				
Median				
Amplitude	0.807	0.641	1.952	0.066
Conduction velocity	1.722	0.641	1.402	0.217
Ulnar				
Amplitude	0.349	0.972	0.789	0.658
Conduction velocity	0.642	0.790	0.482	0.910

Significant values are in bold.

## Discussion

NDTF guidelines require all NCS to be done with fixed distance between stimulator and recording electrodes (80 mm for median and ulnar motor, and 140 mm for median and ulnar sensory)^1,2^. However, we felt that the use of such fixed distances is arbitrary and can cause erroneous NCS results especially in extremes of hand length. For example, sensory median and ulnar NCS in extremely small hands with fixed distance of 140 mm may lead to the recording electrode/stimulator being too proximal in the forearm and the nerve being too deep affecting the NCS parameters. In this context, we endeavoured to see the effects of hand length on NCS parameters in fixed distance vs landmark based NCS through this study.

Our initial hypothesis was disproved in the study. We had postulated that on using a fixed distance stimulation (standard protocol), longer hands will have a distal recording of SNAP and hence larger SNAPs as nerve is superficial distally. However, when a fixed distance of 140 mm was used in our study, no significant variations in sensory amplitudes were found with respect to hand length.

Comparing standard protocol with modified protocol showed that a reduced distance between stimulator and recording electrode (corresponding to a distal recording of SNAP where the nerve is superficial) is associated with larger amplitude of SNAP (Table 1). This is significant in that the two groups were matched in confounders such as age, gender, height and BMI as the same subject underwent both standard and modified protocols (Table 1). Similar findings were noted on direct comparison of shorter and longer hands, where, in modified protocol, longer stimulator to recording electrode distance was associated with smaller SNAP and CMAP amplitudes. However, this difference failed to reach statistical significance in motor NCS and Median sensory NCS (Table 2).One possibility for this could be the fact that the longer hands group had a lesser BMI (25.64 ± 4.0 kg/m^2^) than the shorter hands group (27.49 ± 5.3 kg/m^2^), as BMI is known to significantly alter NCS amplitudes^3^.

Hand length is the distance between tip of middle finger to mid styloid point. This can be further represented as the sum of finger length (tip of finger to palmar-digital crease) and palm length (palmar-digital crease to mid styloid point). Anthropometrically, the base of proximal phalanx seen on dorsal aspect of hand is proximal to the palmar-digital crease. It is therefore possible that finger and palm length may not contribute proportionately to hand length as they are morphologically determined rather than being determined by anatomical joints. As a result, our postulate that longer hands will have a distal recording of SNAP in standard protocol may have been inaccurate, as the distance of the electrodes is measured from palmar-digital crease. This might have led to the failure of our initial hypothesis. Unfortunately, we did not measure the finger length, palm length and distance of recording electrode (in sensory NCS)/stimulator (in motor NCS) from the wrist in this study. Further studies may be needed to throw light on this aspect.

Overall, the study suggests that SNAP amplitudes depend on the distance between the stimulator and recording electrode than on hand length. Landmark based NCS leads to variation in stimulator to recording electrode distance. Standardisation of normative NCS parameters in such scenario would be difficult as it would have to take this variation into account. Using a standard stimulator to recording electrode distance as advised by NDTF negates this variation.

Electrophysiological techniques would be best if least number of factors affect their values. In this study, we endeavoured to see if a landmark based NCS would achieve this goal. To our knowledge, this is the first study to compare the standard fixed distance NCS to a landmark based NCS. Neither the standard protocol nor the modified protocol employed in this study, affected the sensory or motor NCS amplitudes, with respect to hand length. In addition, Ulnar motor conduction velocity was the only factor significantly affected by hand length in both protocols. Median motor conduction velocity, on the other hand, showed a trend towards statistical significance (p values 0.07 and 0.09 in standard and modified protocol respectively). Possible explanation for these findings could be that conduction velocities are more sensitive (than amplitudes) to changes in distance between the stimulator and electrodes. However, it remains to be explored as to why motor but not sensory velocities were affected by hand length. With respect to CMAP/SNAP amplitudes, it is likely that the effects of proximal stimulation/recording (where the nerve is deeper) is compensated by using higher voltage of current stimulation. Therefore, it is theoretically possible that if the nerve is superficial (relatively distal), lesser current is sufficient for stimulation/recording and if the nerve is deep (relatively proximal), higher current is needed for simulation/recording in both standard and modified protocols. As a result, any changes in NCS amplitudes due to hand length may have been negated by the amount of current used in the recording. This may be the reason for hand length not having any effect on NCS parameters (except ulnar motor conduction velocity) irrespective of the protocol used. While we have used supramaximal stimulation for every nerve tested, sadly, the threshold of current needed for obtaining the maximal amplitude of CMAP/SNAP was not studied. Overall, we conclude that the modified protocol did not have any additional advantage to the standard protocol advised by NDTF.

Previous studies have explored the relation between hand length and NCS stimulation intensities^4^. However, the effects of hand length on standard NCS parameters remained unexplored. The fact that effects of hand length on standard NCS parameters, and landmark based NCS were studied for the first time in literature, makes this study important with respect to technical aspects of NCS. A single trained Neurophysiology technician for all the NCS reduced the possibility of technical variations. On the other hand, lack of finger length, palm length and distance of recording electrode (in sensory NCS)/stimulator (in motor NCS) from the wrist are limitations of this study. Also, this study is focussed on the distal nerve segments. Landmark based NCS of proximal segments is not done. The threshold of current needed for recording maximal CMAP/SNAP amplitude and the depth of the nerve at the sites of stimulation/recording are not assessed. Future studies may address these limitations.

## Conclusion

When considering the effects of hand length on NCS, a single parameter (Ulnar motor conduction velocity) was influenced by hand length in both standard and modified protocols. Hand length had similar effects on all the NCS parameters irrespective of the protocol employed. The modified protocol did not have any additional advantage to the standard protocol advised by NDTF. Hence, we conclude that the NDTF guidelines are reasonable.

## Data Availability

Data cannot be shared publicly because of ethical restriction and respect for anonymity. Data are available upon request from Sai Deepak Yaranagula, Dept of Neurology, AIG Hospitals, Hyderabad, India; email-sd1162010@gmail.com.
